# The small heat-shock proteins IbpA and IbpB reduce the stress load of recombinant *Escherichia coli *and delay degradation of inclusion bodies

**DOI:** 10.1186/1475-2859-4-6

**Published:** 2005-02-11

**Authors:** Ha LeThanh, Peter Neubauer, Frank Hoffmann

**Affiliations:** 1 Institute for Biotechnology, Department of Biochemistry/Biotechnology, Martin-Luther-University Halle-Wittenberg, Kurt-Mothes-Str. 3, D-06120 Halle, Germany; 2 Bioprocess Engineering Laboratory, P.O. Box 4300, Department of Process and Environmental Engineering, Biocenter Oulu, University of Oulu, FIN-90014 Oulu, Finland

## Abstract

**Background:**

The permanently impaired protein folding during recombinant protein production resembles the stress encountered at extreme temperatures, under which condition the putative holding chaperones, IbpA/IbpB, play an important role. We evaluated the impact of *ibpAB *deletion or overexpression on stress responses and the inclusion body metabolism during production of yeast α-glucosidase in *Escherichia coli*.

**Results:**

Deletion of *ibpAB*, which is innocuous under physiological conditions, impaired culture growth during α-glucosidase production. At higher temperatures, accumulation of stress proteins including disaggregation chaperones (DnaK and ClpB) and components of the RNA degradosome, enolase and PNP, was intensified. Overexpression of *ibpAB*, conversely, suppressed the heat-shock response under these conditions. Inclusion bodies of α-glucosidase started to disaggregate after arrest of protein synthesis in a ClpB and DnaK dependent manner, followed by degradation or reactivation. IbpA/IbpB decelerated disaggregation and degradation at higher temperatures, but did hardly influence the disaggregation kinetics at 15°C. Overexpression of *ibpAB *concomitant to production at 42°C increased the yield of α-glucosidase activity during reactivation.

**Conclusions:**

IbpA/IbpB attenuate the accumulation of stress proteins, and – at high temperatures – save disaggregated proteins from degradation, at the cost, however, of delayed removal of aggregates. Without *ibpAB*, inclusion body removal is faster, but cells encounter more intense stress and growth impairment. IbpA/IbpB thus exert a major function in cell protection during stressful situations.

## Background

Production of recombinant proteins as inclusion bodies in *Escherichia coli *can induce several stress responses [1-3] and may interfere with primary metabolism and cellular protein synthesis [4-6]. The metabolic burden of the synthesis of recombinant and stress proteins [7,8] may result in stop of the synthesis of components of the protein synthesising machinery, and ribosomes may be actively degraded [4,9,10]. Most commonly, the heat-shock response is induced, as the recombinant proteins – unable to fold properly – overload the chaperone network [11,12]. While appropriate execution of stress responses is of vital importance for the host *Escherichia coli *[13], the high metabolic load of strong foreign protein production can prevent proper adaptation [14,15]. The stress situation can become permanent, and only few cellular proteins except heat-shock proteins are synthesised.

Analogously, incubation at extreme temperatures is characterised by near exclusive heat-shock protein synthesis as a consequence of extensive aggregation of cellular proteins [18,19]. To deal with this challenge, the chaperone DnaK changes its function: Under physiological conditions, DnaK promotes folding of kinetically trapped intermediates by ATP-dependent partial unfolding, but at high temperatures it becomes a holding chaperone and binds its client proteins permanently, unable to release them in a folding-competent conformation [20].

The small heat-shock proteins (sHsps) are intrinsic holding chaperones of multimeric structure. The multimers dissociate at high temperatures into smaller entities for exposure of client binding sites. After binding of misfolded client proteins, the sHsps form large globular assemblies [21]. Thereby, they prevent irreversible aggregation, and under permissive conditions transfer the client proteins to ATP-dependent chaperones for refolding or degradation [22,23]. Also the *E. coli *sHsps, IbpA and IbpB, reversibly dissociate upon prolonged incubation at 50°C from multimers of 2 – 3 MDa to monomers in the case of IbpA and to oligomers of 650 – 700 kDa in the case of IbpB, exposing hydrophobic sites [24,25]. IbpA and IbpB are highly homologous [26], but show distinct features. While IbpB is soluble when overexpressed alone, prevents inactivation or aggregation of proteins upon heat and oxidative stress *in vitro *and facilitates their subsequent refolding by DnaK [23-25], a role for IbpA in mediating the transfer of IbpB and other proteins into the insoluble cell fraction has been proposed [19,27]. IbpA and IbpB have been found associated with thermally aggregated cellular proteins and with inclusion bodies [26,28]. The resolubilisation of protein aggregates, which *in vitro *and *in vivo *is mediated by ClpB together with DnaK [28-31], can be retarded both in strains overexpressing *ibpAB *as well as in some cases in strains lacking *ibpAB *[19,32,33]. IbpA and IbpB might control the reversible transition between soluble and aggregated forms that account for the plasticity of inclusion bodies [27,34,35].

The role of IbpA/IbpB in cell protection is less clear. Overexpression of *ibpAB *confers resistance to heat and other stresses [37], but deletion of the *ibpAB *operon does not affect growth even at high temperatures. Only recovery from prolonged exposure to extreme temperatures such as 50°C are impaired in an *ibpAB *mutant, and growth of *ibpAB dnaK *double mutants at high temperatures [19,36]. In this study we exploit the similarities between incubation at extreme temperature and production of aggregation-protein proteins to examine the functions of the small heat-shock proteins IbpA/IbpB in inclusion body metabolism and stress relief during α-glucosidase production. The enzyme α-glucosidase from *Saccharomyces cerevisiae *produced in recombinant *E. coli *is a suitable model system. It accumulates as inclusion bodies and induces the heat-shock response including prolonged expression of the *ibpAB *operon [17]. It is, however, also able to fold into its native conformation in *E. coli *if produced at low temperature [38], enabling the study of in-vivo-reactivation kinetics.

## Results

### IbpA/IbpB levels during α-glucosidase production with and without *IbpAB *coexpression

The concentrations of IbpA and IbpB were estimated from the proteomes of cells producing α-glucosidase for two hours. The *ibpAB *operon was induced already during α-glucosidase production at 30°C, with moderately higher levels at elevated temperatures (Table 1), whereas no IbpA/IbpB was detected in the plasmid-free strain. The *ibpAB *operon including its own promoter was inserted immediately downstream of the α-glucosidase gene into the plasmid pKK177-3/GLUCP1, expecting overexpression of *ibpAB *from its heat-shock promoter and as a tricistronic operon together with α-glucosidase. This plasmid gave ten and four times higher concentrations of IbpA and IbpB, respectively, at 30°C, and the sHsp level increased further in proportion to temperature (Table 1). With *ibpAB *overexpression, the IbpA accumulation was intensified more strongly and the ratio of IbpA to IbpB rose from 2–3 to 6–8. As the two sHsp may exert different functions, this change need to be accounted for. Depending on the temperature, overexpression increased the concentrations of IbpA and IbpB relative to their concentrations without overexpression four to more than 25 times (Table 1). This wide range of concentrations served to investigate the impact of IbpA/IbpB on α-glucosidase production and in-vivo-reactivation.

### Elevated levels of IbpA/IbpB increase α-glucosidase accumulation

α-Glucosidase was produced in *E. coli *MC4100 with and without *ibpAB *overexpression and in an otherwise isogenic *ibpAB *deletion mutant. α-Glucosidase accumulation was enhanced at elevated IbpA/IbpB level and reduced in the deletion strain in a temperature dependent manner; the effect was strongest at high temperature (Table 2). At temperatures below 30°C, the IbpA/IbpB levels did not influence the amount of α-glucosidase, which accumulated mainly in the soluble cell fraction in active form (Table 2).

**Figure 1:**
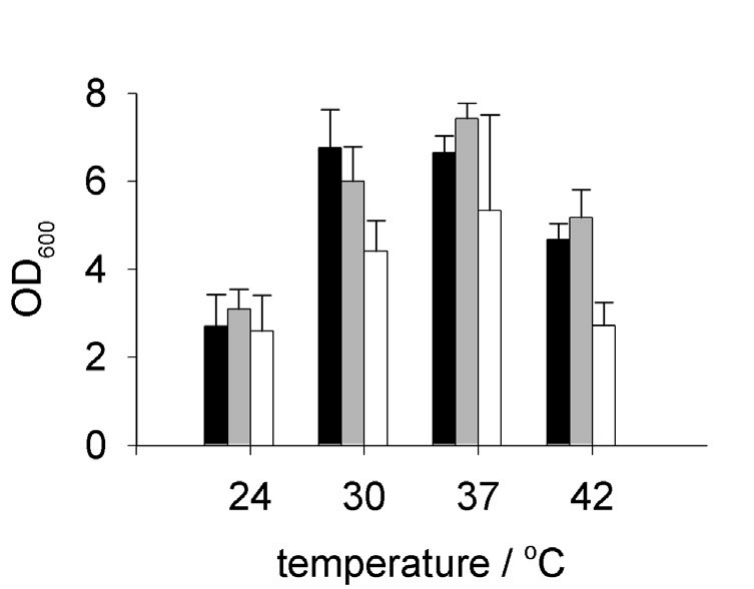
**Impact of IbpA/IbpB levels on growth during α-glucosidase production at various temperatures. **Cultures were grown on complex Luria-Bertani medium at 37°C to an OD_600 _of 0.5 and after induction of α-glucosidase synthesis by addition of 1 mM IPTG incubated at 24°C, 30°C, 37°C, or 42°C. Growth was linear after induction, and optical density OD_600 _values after four hours of α-glucosidase production are shown for wildtype strain with (black bars) or without overexpression of *ibpAB *(grey bars) and the *ibpAB *deletion strain (white bars). Mean values and 95% confidence intervals from at least three independent experiments are shown.

Post-induction growth of the *ibpAB *deletion mutant was retarded at higher temperatures (Fig. 1), although there was no impact of the *ibpAB* deletion on growth without induction of α-glucosidase synthesis (not shown) or concomitant to production at lower temperature.

Thus, the sHsp are effective only under conditions that favour inclusion body formation.

### IbpA/IbpB attenuate accumulation of stress proteins during α-glucosidase production

**Figure 2:**
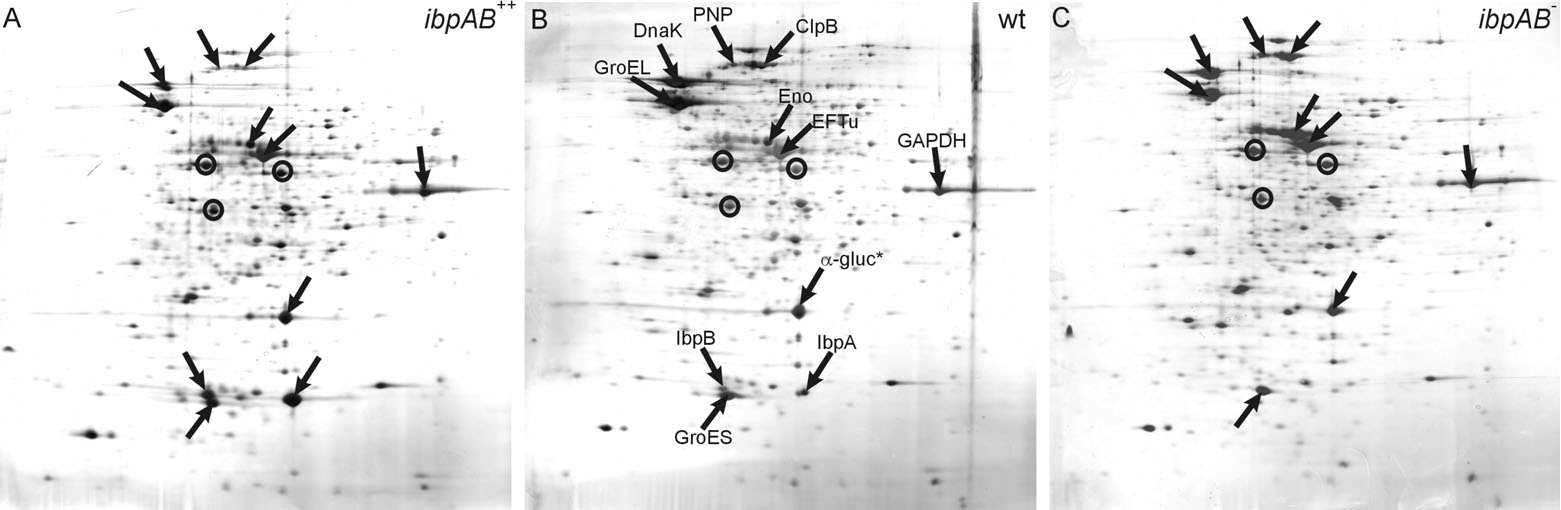
**Effect of *ibpAB *availability on the proteome composition after two hours of α-glucosidase production at 42°C. **After growth at 37°C to OD_600 _of 0.5, cultures were induced with 1 mM IPTG, and transferred to 42°C. Two hours later, cells were harvested and the proteome was separated by two-dimensional gel-electrophoresis. Silver-stained two-dimensional gels of (A) wildtype strain with or (B) without *ibpAB *overexpression, and of (C) the *ibp AB *deletion strain. Reference spots used for normalisation of spot intensities after densitometry are indicated by circles. Positions of selected proteins (cf. text) are indicated.

**Figure 3:**
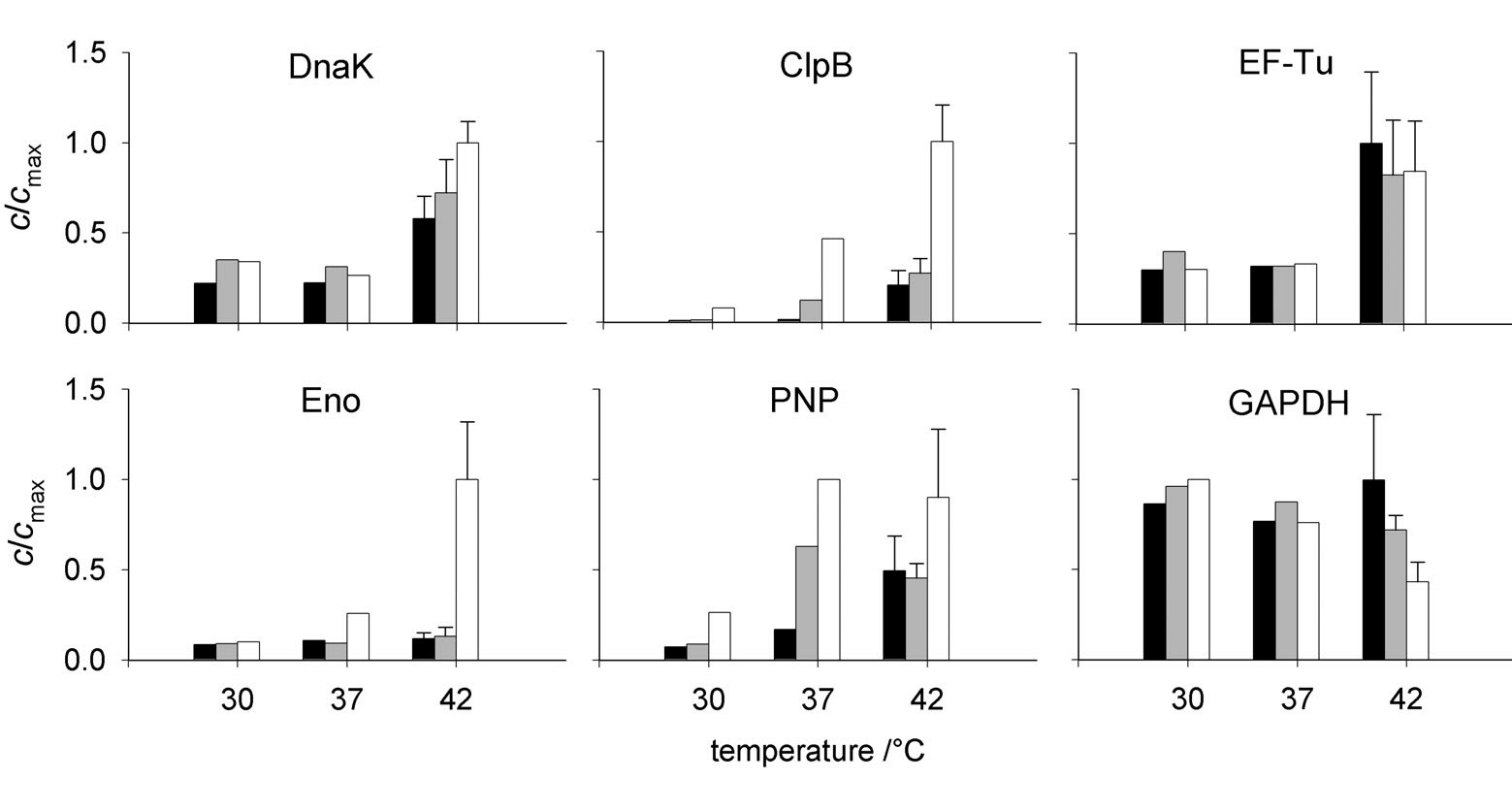
**Level of stress proteins after production of α-glucosidase at different temperatures. **Cultures were grown to an OD_600 _of 0.5 at 37°C, induced with 1 mM IPTG and transferred to the indicated temperatures for two hours. After two-dimensional gel electrophoresis of the proteomes, spot volumes quantified by densitometry of Coomassie-stained gels were normalised with the summed volumes of three reference spots marked in Fig. 2 by circles. Concentrations *c *in the wildtype strain with *ibpAB *overexpression (black bar), without overexpression (grey bar), and in the *ibpAB *deletion mutant (white bar) are given relative to the maximum concentration *c*_max _determined for the respective protein to facilitate comparison. The 95% confidence interval from four experiments at 42°C are shown. Protein designation are given above the panels: chaperones DnaK and ClpB, the thermo-unstable elongation factor (EF-Tu), enolase (Eno), polynucleotide phosphorylase (PNP), and glyceraldehyde 3-phosphate dehydrogenase (GAPDH). Production temperatures are given below the bars.

To study the impact of IbpA/IbpB on cellular reactions during α-glucosidase production, the proteomes of cultures producing α-glucosidase for two hours at various temperatures were separated by two-dimensional gel electrophoresis (shown for 42°C in Fig. 2). While chaperones assisting de-novo protein folding such as GroEL and GroES were not significantly influenced by IbpA/IbpB level (Fig. 2), the DnaK concentration showed a negative correlation with IbpA/IbpB availability at 42°C (Fig. 3). The disaggregating chaperone ClpB reached higher concentrations in the *ibpAB *deletion mutant at all temperatures, in addition to the induction at elevated temperatures (Fig. 3). ClpB accumulation was repressed, however, in the wildtype strain at 30°C, and with overexpression of *ibpAB *both at 30 and 37°C. As the overall level of IbpA/IbpB was low at these temperatures, and other cellular proteins were not affected, this repression is unlikely to be a passive effect of the metabolic burden of *ibpAB *expression.

A similar pattern was found for enolase, which showed higher levels at elevated temperatures only in the deletion mutant (Fig. 3). As enolase is a glycolytic enzyme as well as a putative structural component of the RNA degradosome [39], we examined another metabolic enzyme, glyceraldehyde 3-phosphate dehydrogenase (GAPDH), and polynucleotide phosphorylase (PNP), another RNA degradosome member. The positions on the 2D gels of further degradosome components, RNAse E and RhlB, are not listed in the database. While PNP accumulated to higher concentrations at elevated temperatures and reached highest levels in the deletion mutant (Fig. 3), GAPDH concentration was not significantly affected by temperature and had a slightly lower concentration in the *ibpAB* deletion mutant at 42°C (Fig. 3). Thus, enolase seems to be induced as a part of a stress response rather than as a metabolic enzyme.

A typical house-keeping protein, the translational elongation factor EF-Tu, had a two to three times higher level at 42°C than at lower temperatures, but the concentrations were not effected by IbpA/IbpB level (Fig. 3).

Thus, while housekeeping proteins are not influenced by IbpA/IbpB levels, the accumulation of ClpB and components of the RNA degradosome was intensified in the *ibpAB *deletion mutant and attenuated with *ibpAB *overexpression. This indicates that IbpA/IbpB can relieve the cells of the stress encountered during α-glucosidase production.

### Impact of chaperones on in-vivo-reactivation and degradation of α-glucosidase from inclusion bodies

The impact of chaperones on the metabolism of α-glucosidase inclusion bodies was examined in wildtype *E. coli *MC4100 with or without overexpression of *ibpAB *and in isogenic strains carrying deletions of *ibpAB*, *clpB*, or *dnaK*. After production of α-glucosidase at 37°C for two hours, the cultures were treated with tetracycline to arrest protein synthesis and transferred to 30°C to initiate disaggregation of inclusion bodies as reported for other proteins [34]. In the wildtype and *ibpAB *strains, disintegration of inclusion bodies, increase of α-glucosidase activity, and higher concentrations of α-glucosidase in the soluble cell fraction could be detected already within 20 min; the disaggregation was accompanied by degradation (cf. below). While the concentration of α-glucosidase was 10–25% higher in *dnaK *and *clpB* deletion mutants than in the wildtype strain, the specific activities obtained during the production phase were less than 0.1 Umg^-1^, compared to about 0.3 Umg^-1 ^in the other strains (data not shown). Importantly, neither disaggregation, nor reactivation nor degradation took place in these mutants within two hours, and the concentration of α-glucosidase inclusion bodies remained unchanged.

**Figure 4:**
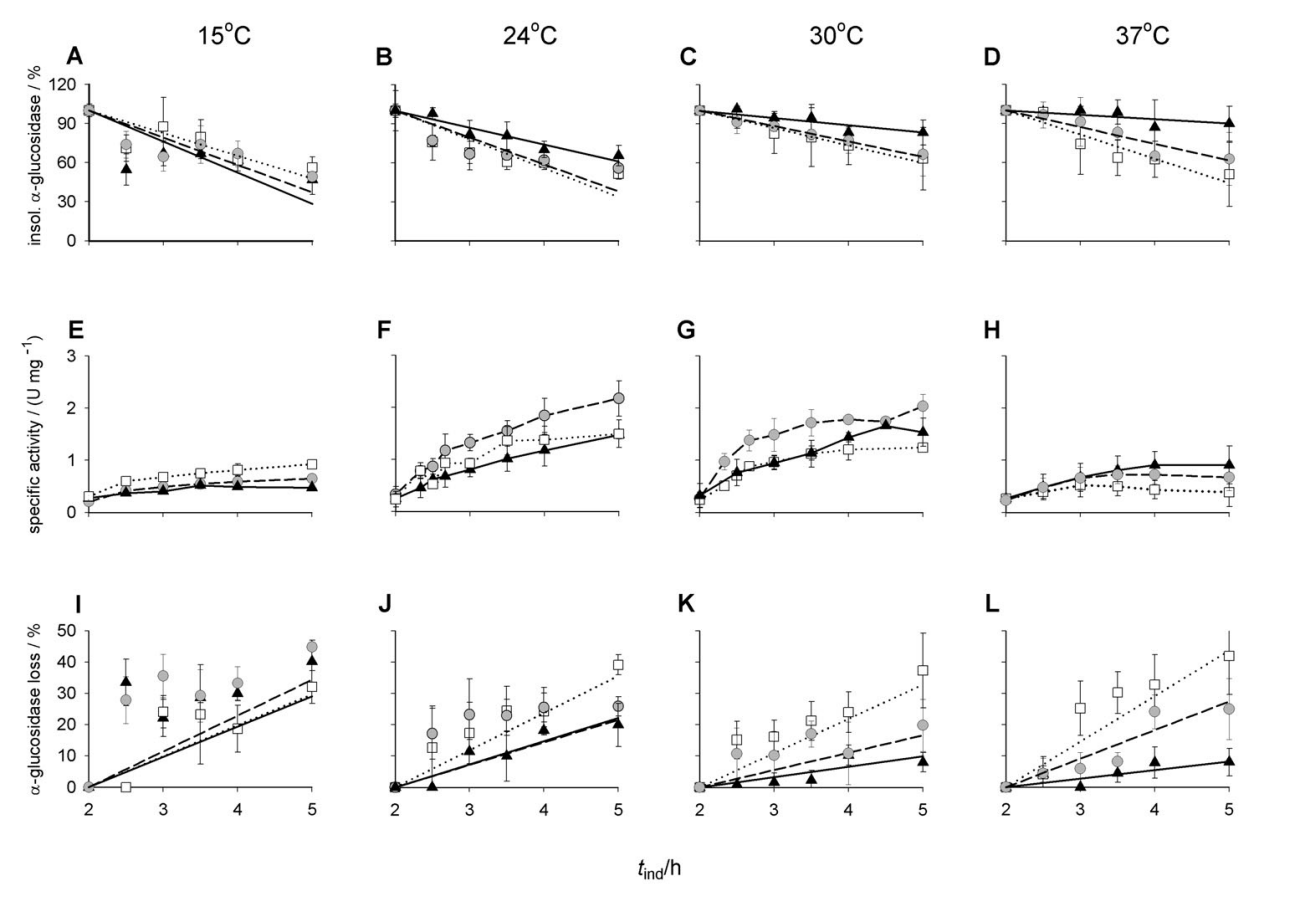
**Kinetics of disaggregation, degradation and reactivation of α-glucosidase from inclusion bodies at various temperature. **Cultures producing α-glucosidase in the wildtype strain with overexpression of *ibpAB *(black triangles, solid lines) or without (grey circles, dashed lines) and in the *ibpAB *deletion mutant (white squares, dotted lines) were incubated at 37°C to an OD_600 _of 0.5 and then induced with 1 mM IPTG. Two hours after induction, tetracycline was added to a final concentration of 25 μg mL^-1^, and the cultures were transferred to 15°C (A, E, I), 24°C (B, F, J), 30°C (C, G, K) or 37°C (D, H, L), respectively. Mean value and confidence interval of at least 2 (15 and 24°C) or 3–5 (30 and 37°C) independent experiments. Lines are from linear regression. (A-D) The amount of insoluble α-glucosidase was estimated by densitometry of Coomassie-stained SDS-PAGE gels and was referred to the value at the time of reactivation start (54 mg g^-1 ^in the wildtype, 58 mg g^-1 ^with overexpression of *ibpAB *and 49 mg g^-1 ^in the deletion mutant). (E-H) α-Glucosidase activity and protein content were determined in cell extracts after disruption. Specific activity of α-glucosidase is given in units per mg of protein. (I-L) Degradational loss of α-glucosidase calculated as difference of the sum of soluble and insoluble α-glucosidase to the value obtained before addition of tetracycline. Time is given in hours after induction.

The kinetics of disaggregation, reactivation and degradation of inclusion bodies at modified levels of IbpA/IbpB, i.e. with deletion or overexpression of *ibpAB*, were investigated as a function of temperature. After production of α-glucosidase at 37°C, the cultures were transferred to various temperatures. As protein synthesis was arrested before the transfer, the initial IbpA/IbpB level did not depend on the disaggregation temperature. The α-glucosidase concentration in the insoluble cell fractions of all cultures decreased with time, but with different kinetics. In the *ibpAB *deletion strain, the disaggregation kinetics were not influenced by the incubation temperature (Fig. 4A–D). With overexpression of *ibpAB*, the removal of α-glucosidase from the inclusion bodies was fastest at 15°C (Fig. 4A), proceeding with similar rates as in the other strains. The disaggregation was, however, progressively slower at 30 and 37°C (Fig. 4C,D). At 37°C, α-glucosidase was largely preserved in the insoluble cell fraction of the *ibpAB *overexpressing strain (Fig. 4D). Thus, disaggregation was retarded at higher temperatures in an IbpA/IbpB dependent manner.

Disaggregation of α-glucosidase inclusion bodies was accompanied by an increase of the specific α-glucosidase activities in the cell extracts. The reactivation showed a clear temperature optimum in the range of 24 to 30°C, most evident with wildtype levels of IbpA/IbpB (Fig. 4F,G). The specific α-glucosidase activities increased sevenfold up to 2 Umg^-1 ^within 3 h, whereas at both higher and lower temperatures of 37°C and 15°C, only 0.5–0.75 Umg^-1 ^were obtained (Fig. 4E,H). Both, *ibpAB *overexpression and deletion, did not improve the maximum reactivation yields. The resolubilisation, measured as an increase of the α-glucosidase concentrations in the soluble cell fraction, showed very similar kinetics as the reactivation (data not shown).

A large part of the disaggregated α-glucosidase was subjected to proteolysis: within three hours after arrest of protein synthesis, 30 – 40% of α-glucosidase were lost due to degradation at 15°C in all three strains (Fig. 4I), and in the *ibpAB *deletion mutant at all temperatures (Fig. 4I–L). Similar to the effect on disaggregation, higher *ibpAB* expression levels or elevated temperatures synergistically retarded degradation, reducing the loss to 10% with *ibpAB *overexpression at 37°C (Fig. 4L). IbpA/IbpB thus influenced disaggregation, resolubilisation, reactivation and degradation in a temperature-dependent manner.

### IbpA/IbpB favour reactivation of α-glucosidase produced at high temperature

**Figure 5:**
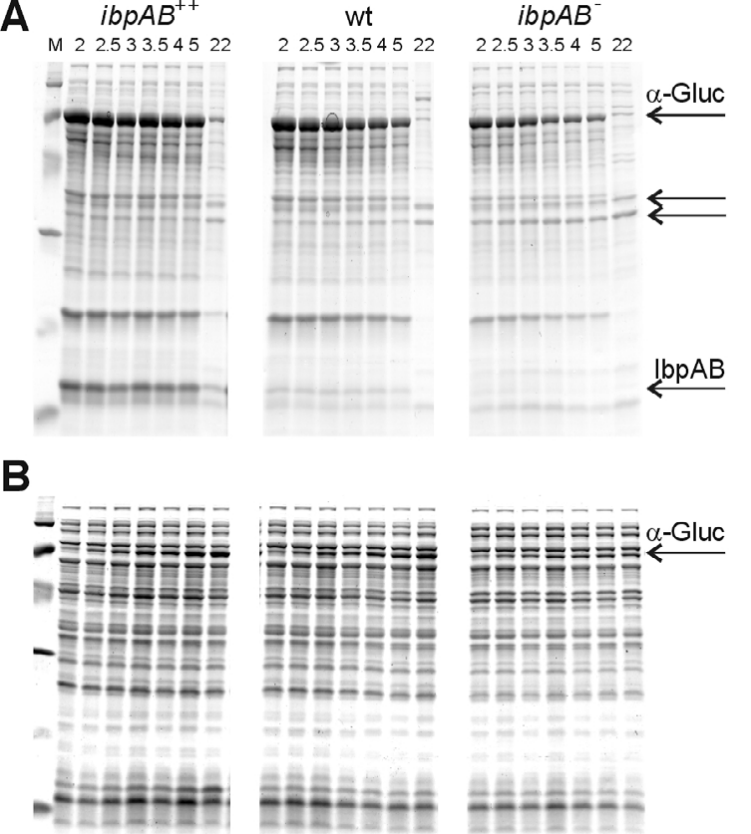
**Disaggregation and resolubilisation of inclusion bodies produced at 42°C **Inclusion bodies of α-glucosidase were produced for two hours at 42°C, followed by addition of tetracycline and subsequent incubation at 30°C. Coomassie-stained SDS-PAGE gel of (A) the insoluble cell fractions and (B) the soluble cell fractions extracted from identical amounts of biomass; times relative to induction and *ibpAB *genotype are given above the lanes. Positions of α-glucosidase, IbpA/IbpB and membrane proteins are indicated. First lane contains molecular weight markers of 97.4, 66.2, 45, 31, 21.5 and 14.4 kDa, respectively.

As IbpA/IbpB are designed for action at extreme temperature, α-glucosidase inclusion bodies were produced at 42°C, followed by arrest of protein synthesis and incubation at 30°C. The production temperature did not influence the initial disaggregation kinetics (data not shown). About one third of the α-glucosidase produced concomitant to *ibpAB *overexpression was resistant to disaggregation after incubation over night, while it was eliminated apart from traces in the other strains (Fig. 5A). Also the removal of other components of the inclusion bodies, which appear as smear in the SDS PAGE of the insoluble cell fractions, were impaired in the overexpressing strain (Fig. 5A), resembling the core inclusion bodies that remain with other proteins [34], whereas the inclusion bodies produced at low IbpA/IbpB levels were completely dissolved after prolonged incubation. Only outer membrane proteins (indicated by arrows) that result from coprecipitation of cell debris during inclusion body preparation [40] remain at constant level in the insoluble cell fraction (Fig. 5A). Thus, the delay of disaggregation by IbpA/IbpB is not specific to α-glucosidase, but effects also cellular proteins in a similar way.

**Figure 6:**
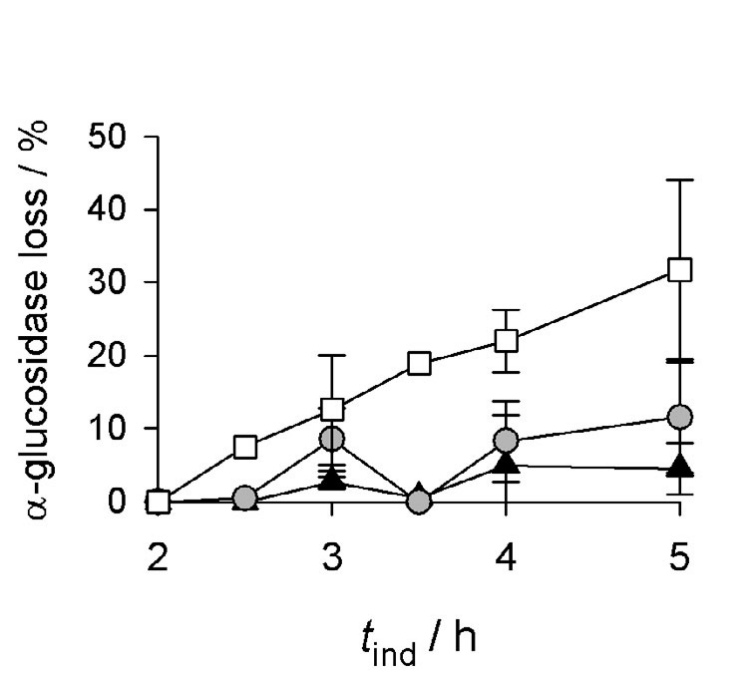
**Degradation of α-glucosidase after production at 42°C. **Cultures of *ibpAB *deletion mutant (white square), wildtype strain without (grey circle) or with *ibpAB *overproduction (black triangle) produced α-glucosidase for two hours at 42°C and were transferred to 30°C after addition of tetracycline. Degradational loss of α-glucosidase is shown relative to the amount of α-glucosidase present at the time of tetracycline addition. Time is given in hours after induction.

Production at 37°C resulted in degradation of 20% of α-glucosidase during incubation at 30°C for 3 h in the wildtype strain (Fig. 4K). After production at 42°C, however, there was less than 10% of α-glucosidase lost during reactivation at 30°C with or without *ibpAB *overexpression (Fig. 6). Thus, degradation of α-glucosidase after arrest of protein synthesis was predetermined by the preceding production temperature. The protection was dependent on IbpA/IbpB availability, as in the deletion mutant the loss of α-glucosidase was substantial regardless of the production temperature (Fig. 4K, 6).

Consequently, disaggregated α-glucosidase was quantitatively transferred to the soluble cell fraction after production at 42°C concomitant to *ibpAB *overexpression, and became the most prominent band in the gel. The band intensity increased from 2 to 7% of the total cellular protein (Fig. 5B). In contrast, significantly less α-glucosidase could be detected in the soluble fraction of the deletion mutant even after prolonged incubation, finally corresponding to only 4% of cellular protein (Fig. 5B).

**Figure 7:**
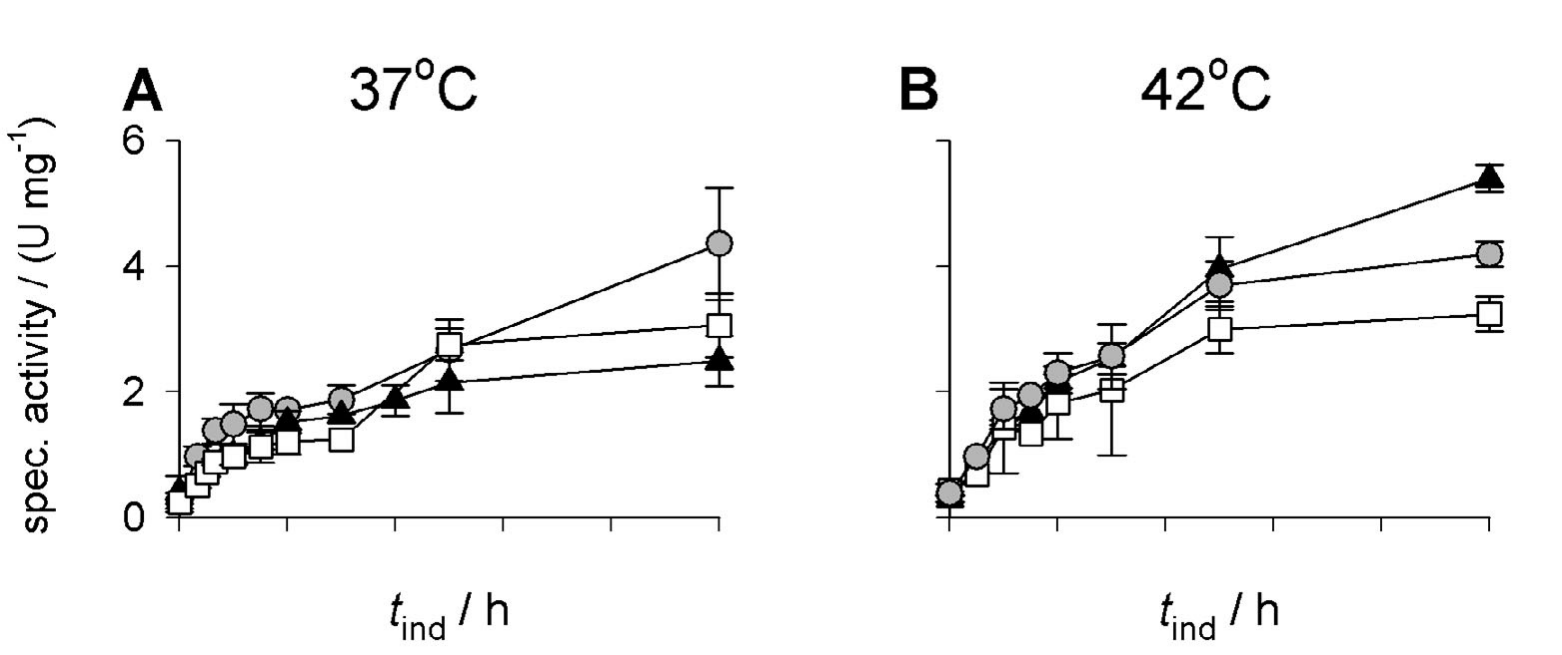
**Impact of production temperature on reactivation of α-glucosidase from inclusion bodies. **Inclusion bodies of α-glucosidase were produced for two hours at 37°C (A) or 42°C (B) in *ibpAB *deletion mutant (white square), wildtype strain without (grey circle) or with *ibpAB *overproduction (black triangle). Specific activity of α-glucosidase was measured during incubation at 30°C after addition of tetracycline. Mean values and 95% confidence intervals from six (37°C) or four (42°C) independent experiments. Time is given in hours after induction.

The α-glucosidase activity increased during the initial reactivation phase with similar slopes with and without *ibpAB *overexpression (Fig. 7B). Stationary level of activity was reached earlier, however, in the *ibpAB *deletion mutant and in the wildtype strain without overexpression. Hence the final activities in these strains were independent of the production temperature (Fig. 7A,B). In contrast, the activity continued to increase for about 12 h after arrest of protein synthesis subsequent to α-glucosidase production at 42°C with *ibpAB *overexpression (Fig. 7B), resulting in higher activity yields than obtained in the wild type strain. The final α-glucosidase activities after production with overexpression of *ibpAB *at 42°C were two times higher than after production at 37°C (Fig. 7A,B). IbpA/IbpB can thus ameliorate reactivation of inclusion bodies produced at high temperature.

## Discussion

### Intensified stress response and growth impairment in *ibpAB *deletion strain

Impairment of cell growth by *ibpAB *deletion is observed only under conditions characterised by depletion of free DnaK, such as under extremely high temperature or artificially controlled low level [19,33,36,37]. Accumulation of α-glucosidase as inclusion bodies mimics the aspect of permanently impaired protein folding, and growth impairment during α-glucosidase production was more intense in the *ibpAB *mutant. Moreover, DnaK controls the expression of heat-shock genes. Consequently, chromosomal *ibpAB *and other heat-shock genes were induced during α-glucosidase production. Especially in the deletion strain, lack of IbpA/IbpB was compensated by enhanced accumulation of the disaggregating chaperone ClpB. Also the levels of two components of the RNA degradosome, enolase and PNP, were elevated in the *ibpAB *deletion strain at 42°C. Enolase homologues have been reported to be induced by heat shock in a variety of species, including *Bacillus subtilis *[41], but there was no indication that the *E. coli eno *gene can be induced by heat-shock or high temperature [42].

### Both ClpB and DnaK are essential for disaggregation and de novo protein folding

ClpB and DnaK accumulated to higher levels in the *ibpAB *deletion strain. These chaperones cooperate with IbpA/IbpB in a "disaggregation triad" [33]. Each chaperone, ClpB and DnaK, was essential for reactivation as well as degradation of α-glucosidase, indicating that both processes operate after disaggregation of the inclusion bodies. With other protein aggregates, varying dependencies on DnaK and ClpB for degradation or reactivation were observed [29,30,32,43-45]. To the aggregate-specific factors influencing the chaperone demand for disaggregation *in vitro *belong their sizes and accessible surface areas [31,46,47]. Also the substrate specificity of chaperones may influence the protein-specific requirements.

The impaired disaggregation in *clpB *and *dnaK *mutants reduced the accumulation of active α-glucosidase already during production, and a negative impact of the *clpB *deletion on *de novo *folding has been observed also with a number of other proteins [48]. Resolubilisation from aggregates might thus be a general route to active protein, in accordance with a quantitative model of inclusion body formation kinetics [16]. This constitutes a major difference to the irreversible aggregation at extreme temperatures, when the client release of DnaK is retarded [20].

### IbpA/IbpB disburden DnaK and reduce degradation

Ongoing attempts to fold α-glucosidase will finally result in its degradation. IbpA/IbpB, which are postulated to recognise similar traits of aggregation prone proteins as DnaK [19], will withdraw α-glucosidase from the futile cycle of DnaK binding and release. Thereby, IbpA/IbpB on the one hand disburden the DnaK system to make resources available to repress stress responses, and on the other hand protect α-glucosidase from degradation. These functions might be related, as the heat-shock response includes also proteases that may accumulate to lower levels with *ibpAB *overexpression. Deletion of *ibpAB*, conversely, lowers the amount of α-glucosidase and other recombinant proteins [27,49]. Consequently, unlike to incubation at 50°C, *ibpAB *deletion did not increase aggregation during α-glucosidase production, rather reduced accumulation of insoluble α-glucosidase. Functional replacement of DnaK by IbpA/IbpB is also reflected by the amount of DnaK associated with inclusion bodies and aggregated cellular proteins, that is increased in the *ibpAB *deletion mutant and decreased by *ibpAB *overexpression [[19], unpublished results].

### IbpA/IbpB prevent degradation in a temperature-dependent manner

Disaggregation and degradation of inclusion bodies took place after arrest of protein synthesis. The disaggregation at and above 30°C was retarded by high levels of IbpA/IbpB. In wildtype cells, IbpA/IbpB accumulation is therefore restricted to requiring conditions, e.g. by degradation of IbpA/IbpB after dissociation from protein aggregates [33] or repression of *ibpAB *expression despite an otherwise active heat-shock response [14].

Temperatures during both, formation and disaggregation of inclusion bodies, were important for the rate of disaggregation and yield of reactivation. Irreversible structural changes of IbpA/IbpB at low temperatures [25] may impair the protective function, making the disaggregation kinetics at 15°C independent of the IbpA/IbpB level. Hence with other systems, depending on the conditions, no influence of the *ibpAB* deletion on disaggregation kinetics or even delayed disaggregation in the mutant were observed [19,33,34,50]. The reactivation rate is determined by the difference of disaggregation and degradation rates, which are influenced in parallel by the availability of the sHsps. Hence reactivation was hardly effected by *ibpAB *deletion or overexpression, in accordance with observation for other proteins [32,34].

With high temperature during production, degradation during the subsequent disaggregation phase was nearly completely avoided in *ibpAB *proficient strains. In the *ibpAB *deletion strain, on the contrary, production temperature did not ameliorate the recovery of soluble α-glucosidase. Consequently, the reactivation phase was prolonged with higher IbpA/IbpB concentrations, and the final α-glucosidase activities were increased from 4 in the wildtype cells to 5 Umg^-1 ^with *ibpAB *overexpression. As the yield of reactivated protein is more important for heat-stress survival than the rate of disaggregation [56], the reduction of degradation may also contribute to the cell protective function of the sHsps.

## Conclusions

Deletion of *ibpAB *renders *E. coli *more susceptible to growth impairment by production of α-glucosidase. Without recombinant protein production, *ibpAB *deletion affects growth only at extreme temperatures or in combination with mutations of *dnaK*. Thus, production of an aggregation prone protein constitutes a good model system to study the functions of IbpA/IbpB under non-lethal conditions. Two major functions were found for IbpA/IbpB: First, the sHsps protect cells during α-glucosidase production and disburden the DnaK system. In the *ibpAB *deletion strain, insufficient free DnaK was available to control ClpB accumulation, which was strongly intensified. Also enolase and PNP showed a similar pattern.

Second, IbpA/IbpB reduce degradation of α-glucosidase during disaggregation from inclusion bodies. The IbpA/IbpB function depends on elevated temperature. Improved reactivation and protection from degradation were found after production at higher temperature, in accordance with the known heat-activation of sHsps. Also during disaggregation, a higher temperature was beneficial, whereas no protection from degradation was found at 15°C. In the *ibpAB *deletion strain, however, removal of inclusion bodies was accelerated, possibly due to the higher levels of ClpB and DnaK that were found to be essential for reactivation and degradation of α-glucosidase. Thus, the sHsp level is in a delicate balance: IbpA/IbpB are required to protect the cell, but abundant IbpA/IbpB impede the necessary adaptation and retards removal of aggregates.

## Methods

### Strains and Plasmids

*Escherichia coli *MC4100 *araD139 Δ (argF-lac)U169 rpsL150 relA1 flbB5301 deoC1 ptsF25 rbsR *and JGT17 MC4100 Δ *ibpAB *[36] were used to produce the yeast α-glucosidase encoded on the plasmid pKK177-3/GLUCP1 [38]. Plasmid pUBS520, supplying the minor *argU *tRNA and carrying the *lacI *repressor gene [51] was cotransformed for improving the production of α-glucosidase [52]. The sequence from plasmid pIbp [36] between the two *Hind*III restriction sides, coding for the *ibpA *and *ibpB *genes including their native promoter, was cloned into the *Hind*III site of the plasmid pKK177-3/GLUCP1 [38], located between the α-glucosidase *GLUCPI *gene and the 5ST1T2 terminator, to give the new plasmid named pKK177-3/GLUCP1_ibpAB, in which the *ibpAB *operon is under the control of its native promoter and of the *tac*-promoter upstream of the α-glucosidase gene. Proper orientation of the insert was tested by *Ear*I digestion.

### Culture conditions

The cultures were incubated on Luria-Bertani (LB) medium, supplemented with appropriate antibiotics (ampicilline 100 μg mL^-1^, chloramphenicol 50 μg mL^-1^), on a rotary shaker at 37°C to an OD_600 _of 0.5, induced with 1 mM IPTG and transferred to different temperatures as indicated in the results section. For reactivation experiments, tetracycline was added to a final concentration of 25 μgmL^-1 ^two hours after induction to arrest protein synthesis, and the cultures were transferred to the indicated temperatures.

### Product analysis

For SDS-PAGE analysis, cell pellets resuspended in phosphate buffer pH 7 were incubated on ice with 33 mgL^-1 ^lysozyme for 30 min and disrupted by sonication for 20 s. Soluble and insoluble fractions were separated by centrifugation for 20 min at 13,000 rpm. The insoluble fractions were washed with phosphate buffer twice. SDS-PAGE analysis was performed according to the method of Laemmli [53]. Levels of α-glucosidase were estimated by densitometry of Coomassie-stained gels.

α-glucosidase activity was measured as described [54]. For the calculation of specific activities, the protein concentration was determined according to Bradford [55] with bovine serum albumin as standard. Specific activities are expressed as units of α-glucosidase per milligram of total protein (U mg^-1^).

### Two-dimensional gel electrophoresis

Cell pellets from a culture volume of 8/OD_600 _mL were dissolved in 200 μL lysis buffer containing 8 M urea, 4% CHAPS, 60 mM DDT, 2% Pharmalyte 3–10 and 0.002% bromophenol blue and incubated for one hour at room temperature. The first dimension was carried out on an IPGphor using 24 cm pH gradient 3–10 strips (amersham pharmacia biotech), with the voltage profile recommended by the manufacturer. The second dimension was run in an Ettan Dalt six (amersham pharmacia biotech) on 12.5% polyacrylamide gels. Gels were stained with Coomassie brilliant blue for quantification or by silver staining for visualisation. Gels for cultures incubated at 42°C were performed in quadruplicate, for other temperatures once. Protein spots were identified by comparison with the *E. coli *2D database . Spot identities were verified by N-terminal sequencing for ClpB (RLDRLTN) and enolase (SKIVKII). α-glucosidase was not dissolved from the inclusion bodies and is not visible in the gels.

## Figures and Tables

**Table 1:** Relative levels of IbpA and IbpB after two hours of α-glucosidase production at various temperatures with and without *ibpAB *overexpression from plasmid

	Temperature	wildtype	*ibpAB*^++^
IbpA^a^	42°C	3.3 ± 1.3	84 ± 9.5
	37°C	5.0	50
	30°C	1.6	19

IbpB	42°C	1 ± 0.3	12 ± 1.3
	37°C	1.4	7
	30°C	0.7	3

^a ^The level of IbpA and IbpB was estimated by densitometry of Coomassie-stained two-dimensional gels. Spot volumes were normalised using three spots as internal standards (indicated by circles in Fig. 2) and are given relative to the value of chromosomally encoded IbpB at 42°C.

**Table 2:** Effect of temperature and IbpA/IbpB on accumulation and activity of α-glucosidase four hours after induction

	Genotype	24°C	30°C	37°C	42°C
Total α-glucosidase/ (mg g^-1^)	*ibpAB*^++^	38 ± 5	52 ± 1	66 ± 11	47 ± 11
	wt	39 ± 2	47 ± 5	56 ± 16	38 ± 7
	*ibpAB*^-^	36 ± 4	43 ± 2	49 ± 10	28 ± 4

Vol. activity/ (U mL^-1^)	*ibpAB*^++^	0.56 ± 0.17	0.80 ± 0.29	0.13 ± 0.04	0.15 ± 0.01
	wt	0.61 ± 0.14	0.76 ± 0.26	0.10 ± 0.02	0.10 ± 0.01
	*ibpAB*^-^	0.55 ± 0.02	0.74 ± 0.20	0.11 ± 0.04	0.10 ± 0.02

Mean values and 95% confidence intervals from at least three independent experiments. The variance between experiments is mainly caused by different times needed to reach the set temperature after induction depending on the volume of the cultures.
